# The Cancer Problem

**Published:** 1916-03-04

**Authors:** 


### THE CANCER PROBLEM.

The cancer problem can hardly be presented in a brief article, and nothing here is proposed more than an allusion to its existence and to the industry and ingenuity which are constantly applied to its solution. Apart from its practical bearing on human happiness, the question is one of high scientific fascination. Of its complexity and difficulty some estimate may be formed when recollection is made of the number of workers and formidable apparatus of inquiry which have been devoted to its study. Yet, in spite of everything, the secret has so far defied all attempts to penetrate it. The most elaborate and extensive laboratory investigations, supported at great expense and furthered by experiments on a large scale, have no doubt done something at least on the negative side. They have excluded certain suggestions and theories which have been presented at various times with a greater or less measure of support; and, still more, they have established certain facts which may yet prove of high importance. But they have not told us either what cancer is as a biological process, or how it can be prevented or cured. For this reason in part, and partly on other grounds, some have held that laboratory search has exhausted itself, or that it is not from this direction that practical light and aid are to be expected. Not unnaturally, the operating surgeon develops his own view of the question, and more than one valuable and interesting contribution to the natural history of the subject has come from the surgical wards. But the end is always this—there is no hope save in operation, early and prompt and thorough. This may be the practical position, but it is satisfactory neither to patients nor to medical science. At the best, a surgical operation, and more especially a mutilating operation, is a confession of failure—of failure to prevent or, failing this, to cure by some method which is not repellent both to the patient and to the healer.

Of the "cures" which have been vaunted, both within and without the profession, there is, indeed, a lengthy and varied list. Some have excited a pathetic faith, which has, however, not long survived the severe tests of experience. Some, again, have been utilised as the opportunity for gross quackery and fraud. Not one stands to-day as commanding any considerable body of competent opinion, and he would be a bold practitioner who would dare to tell his patient that any particular medicinal method could safely be tried as an alternative to operation. The position is somewhat different when operation is recognised to be impossible. Admittedly, there is no known remedy in which confidence may be placed. Hence it is not an unreasonable thing that any measure, provided it will not do harm, should be tried. This position is sometimes put to a medical practitioner, and it is not easy for him to give a decision. If he assents to methods which have no professional guarantee he runs the risk of seeming to countenance ignorance and pretence. On the other hand, if he forbids such a proposal he may deny the patient the conviction that everything possible has been done. Between these two he may hesitate, yet we fancy that in most instances the solace of the sufferer will be put in front of any personal *amour propre* or misjudged professional dignity.

